# Square-wave voltammetry of human blood serum

**DOI:** 10.1038/s41598-023-34350-1

**Published:** 2023-05-25

**Authors:** Pavlinka Kokoskarova, Leon Stojanov, Kosta Najkov, Natasha Ristovska, Tatjana Ruskovska, Sławomira Skrzypek, Valentin Mirceski

**Affiliations:** 1 Faculty of Medical Sciences, Goce Delcev University, Krste Misirkov 10A, 2000 Stip, Republic of North Macedonia; 2 Institute of Chemistry, Faculty of Natural Sciences and Mathematics, “Ss Cyril and Methodius” University in Skopje, P.O. Box 162, 1000 Skopje, Republic of North Macedonia; 3 Department of Inorganic and Analytical Chemistry, University of Lodz, Tamka 12, 91-403 Lodz, Poland; 4 Research Center for Environment and Materials, Macedonian Academy of Sciences and Arts, Bul. Krste Misirkov 2, 1000 Skopje, Republic of North Macedonia

**Keywords:** Biochemistry, Biochemistry, Electrochemistry

## Abstract

A study on voltammetric analysis of blood serum diluted in a phosphate buffer is presented using advanced square-wave voltammetry at an edge plane pyrolytic graphite electrode. The results demonstrate that even in a complex medium like human blood serum, electrochemical characterization can be achieved through the use of advanced voltammetric techniques in conjunction with an appropriate commercially available electrode, such as the edge plane pyrolytic graphite electrode, which boosts superior electrocatalytic properties. Without undergoing any chemical treatment of the serum sample, the square-wave voltammetry technique reveals, for the first time, the electrode reactions of uric acid, bilirubin, and albumin in a single experiment, as represented by well-defined, separated, and intense voltammetric signals. All electrode processes are surface-confined, indicating that the edge plane sites of the electrode serve as an ideal platform for the competitive adsorption of electroactive species, despite the extensive chemical complexity of the serum samples. The speed and differential nature of square-wave voltammetry are crucial for obtaining an outstanding resolution of the voltammetric peaks, maintaining the quasi-reversible nature of the underlying electrode processes, while reducing the impact of follow-up chemical reactions that are coupled to the initial electron transfer for all three detected species, and minimizing fouling of the electrode surface.

## Introduction

Voltammetry is a powerful tool for investigating the electrochemistry of a wide range of species, and it is widely used in fields such as pharmacy, medicinal chemistry, biochemistry, and other life sciences^1^. The popularity of voltammetry can be attributed to its ability to provide intrinsic information on the energetic, kinetic, and mechanistic aspects of electron and ion transfer reactions through simple experimental setups and low-cost instrumentation. Cyclic voltammetry (CV) is the most commonly used voltammetric technique^1,2^ for mechanistic and kinetic analysis, while advanced pulse-voltammetric techniques are favoured for analytical determinations due to their superior analytical sensitivity^1–3^. Square-wave voltammetry (SWV)^4^, combines the advantages of cyclic and pulse voltammetry^1–3^, while offering enhanced analytical performance and the ability to provide both mechanistic and kinetic information of electrochemical processes^3,4^.

Despite its advantages, the application of voltammetry in complex media such as blood serum, which contains numerous redox-active and surface-active species, poses a challenge^5,6^. However, direct voltammetric determination of biomolecules in human serum is crucial for the development of simple and cost-effective electrochemical biosensors^7^. In previous studies, direct detection of biomolecules in human serum has been performed using surface-modified working electrodes^7^, most frequently featuring complex composite structures designed for electrocatalysis^8^, or immobilized enzymes for selective and sensitive detection of a specific species^9^. However, studies involving bare unmodified electrodes for detection of single or multiple biomolecules are rare^10^.

The current study highlights the potential of voltammetric characterization of blood serum through a deliberate selection of the working electrode and the voltammetric technique. For the first time, it is shown that a SW voltammetric experiment conducted under optimized conditions can effectively characterize electrochemically the presence of uric acid (UA), bilirubin (BR), and albumin (ALB) in blood serum.

A comprehensive literature review reveals that various electrochemical sensors for UA detection have been developed^11–20^ including electrode modifications with poly(procaterol)^11^, N,N-dimethylformamide^12^, ferrocene/Cu_2_O nanoparticles and uricase^13^, mercaptobenzimidazole^14^, nafion membrane, ferrocene and uricase^15^, sulphur-adlayer-coated gold electrode^16^, and cysteamine^17^. Direct determination of UA has been reported using boron-doped diamond microelectrodes in urine samples only^18^. The major challenge in designing an electrochemical amperometric sensor for UA detection is the presence of interferences from dopamine^11^ and ascorbic acid^12^.

Regarding bilirubin, which is a by-product of haem catabolism, a small number of non-enzymatic electrochemical sensors have been developed so far^21–23^. A thorough review^24^ indicates that most amperometric biosensors for bilirubin detection are based on bilirubin-specific enzymes, such as bilirubin oxidase^25–30^, in combination with 3-mercaptopropionate ^24^, carbon nanotubes^25–29^, zirconia and silica nanoparticles^30^.

Albumin (ALB) is the most abundant protein in human plasma, playing a crucial role in regulating the oncotic pressure in blood vessels and serving as a transporter of various biomolecules. An elevated level of urinary albumin above the reference limit can indicate various pathological conditions, including kidney diseases and heart diseases such as heart failure.

Despite the limited number of reports in the literature, direct electrochemical detection of ALB in human serum is possible^31,32^. For instance, an amperometric sensor was designed using an antibody specific for human albumin immobilized on a gold working electrode^31^. Another voltammetric biosensor was developed through the co-polymerization of acrylamide with N, N′-methylenebis-(acrylamide) in the presence of chitosan and ferrocene as a redox mediator^32^.

The objective of the current study is to investigate in detail the voltammetric response of blood serum using advanced, sensitive, and fast voltammetric techniques, such as SWV. The aim is to provide a basis for the qualitative detection of some blood serum components without previous separation steps and without the need for complex electrode surface modification.

## Experimental

The voltammetric measurements were conducted using the PalmSens2 potentiostat/galvanostat (PalmSens BV, Netherlands), which was controlled by PSTrace 4.8 software (PalmSens BV) at room temperature. The measurements were carried out in a conventional three-electrode voltammetric cell that consisted of a range of working electrodes including an Edge-plane pyrolytic graphite electrode (EPGE) with a geometrical surface area of 7.069 mm^2^ (ALS Co., Ltd.), a basal-plane pyrolytic graphite electrode (BPGE) with a geometrical surface area of 7.069 mm^2^ (ALS Co., Ltd.), a glassy carbon electrode (GCE) with a geometrical surface area of 7.069 mm^2^ (ALS Co., Ltd.), a Pt electrode with a geometrical surface area of 7.069 mm^2^ (ALS Co., Ltd.), and a paraffin impregnated graphite electrode (PIGE) with a geometrical surface area of 19.635 mm^2^. An Ag/AgCl electrode (3 mol/L KCl) was used as a reference electrode, while a graphite rod served as an auxiliary electrode. The GCE and Pt electrodes were cleaned using an Al_2_O_3_ slurry and rinsed with water before being dried in the air. The EPGE, BPGE and PIGE electrodes were cleaned by abrasive paper, Al_2_O_3_ slurry, and an ultrasonic bath, followed by rinsing with water and air-drying.

A phosphate buffer solution (pH = 7.34; 0.1 mol/L) was employed as the supporting electrolyte, unless otherwise specified, in which human serum samples were dissolved and analysed. The utilization of human serum samples was approved by the Ethical Committee of the Faculty of Medical Sciences, Goce Delcev University, Stip, through Decision No. 0801–2/13 dated February 16, 2022. The stock solutions of uric acid (Reanal Budapest, p.a. > 99%) and bovine serum albumin (Sigma Aldrich, ≥ 98%) were prepared by dissolving in water, while the bilirubin stock solution (Alfa Aesar, 97%) was first dissolved in a few drops of dimethyl sulfoxide (Thermo Scientific, 99.7%) before water was added to attain the final desired concentration. All solutions were prepared using water from the Arium® mini Plus purification system provided by Sartorius. Data presented in this study were obtained from 17 human serum samples. The procedure for serum preparation was consistent with established protocols, which involved collecting whole blood and allowing it to coagulate in the presence of a clot-activator for 15–25 min at room temperature. The formed clot was then removed through centrifugation at 1000–2000 rotations/minute for 10 min, resulting in the separation of the serum as a supernatant fraction. The obtained serum samples were stored at − 5 °C without further treatment and were used as required. Some of the samples were obtained from patients with medical conditions, resulting in elevated concentrations of the analysed analytes.

The serum concentrations of uric acid, bilirubin, and albumin were determined using diagnostic reagents from BioSystems (China) and analysed on a clinical chemistry analyser (BS-240Pro, Mindray, Spain) immediately after the routine procedures of blood collection and serum separation.

The spectrophotometric method based on the uricase enzyme was used for the determination of serum uric acid. The method involves the formation of hydrogen peroxide, which reacts with 2, 4-dichlorophenol sulfonate and 4-aminoantipyrine in the presence of peroxidase, leading to the formation of a red-colored quinoneimine dye complex.

For the determination of bilirubin in serum, a spectrophotometric method based on diazo reaction was employed. Direct bilirubin, which is water-soluble, can be directly determined by its reaction with diazotized sulfanilic acid. Indirect, or unconjugated bilirubin, which is bound to albumin, requires the removal of albumins for determination of its concentration, for which the use of an accelerator (cetrimide) is necessary.

The determination of serum albumin was performed using a colorimetric method based on the reaction between albumin in the sample and bromocresol green. In an acidic medium, the binding of albumin to bromocresol green leads to a change in the absorbance of the complex that is proportional to the albumin concentration.

## Methodology

This scientific research study was conducted through a partnership between State University “Goce Delcev”-Stip and private health facility “Primarius d-r Samardziski”-Stip, based on a memorandum of cooperation.

The research, which involves the use of human biological material (serum), was approved by the Ethics Committee for Research at Faculty of Medical Sciences, Goce Delcev University, Stip, involving human subjects under protocol number 0801-2/13 on February 16th, 2022.

### Statement

All experiments and methods were performed in accordance with relevant guidelines and regulations.

Informed consent was obtained from all subjects, and all methods were carried out in accordance with the relevant guidelines and regulations of the Ethical Committee of the Faculty of Medical Sciences, Goce Delcev University, Stip involving human subjects under protocol number 0801-2/13 on February 16th, 2022.

The authors confirm that all research was conducted in accordance with relevant regulations and declare that consent was obtained from all participants. Venous blood was collected from both healthy subjects and patients with various pathologies, within the appropriate age group. The volunteers were fully informed about the experimental aspects of the study prior to their participation. Serum was then isolated from the collected blood samples.

All participants who signed their consent were included in the study, while those who did not respond affirmatively to the questionnaire were excluded. The respondents were assured of anonymity in both the experimental process and the publication of results for scientific purposes.

## Results

**Figure 1:**
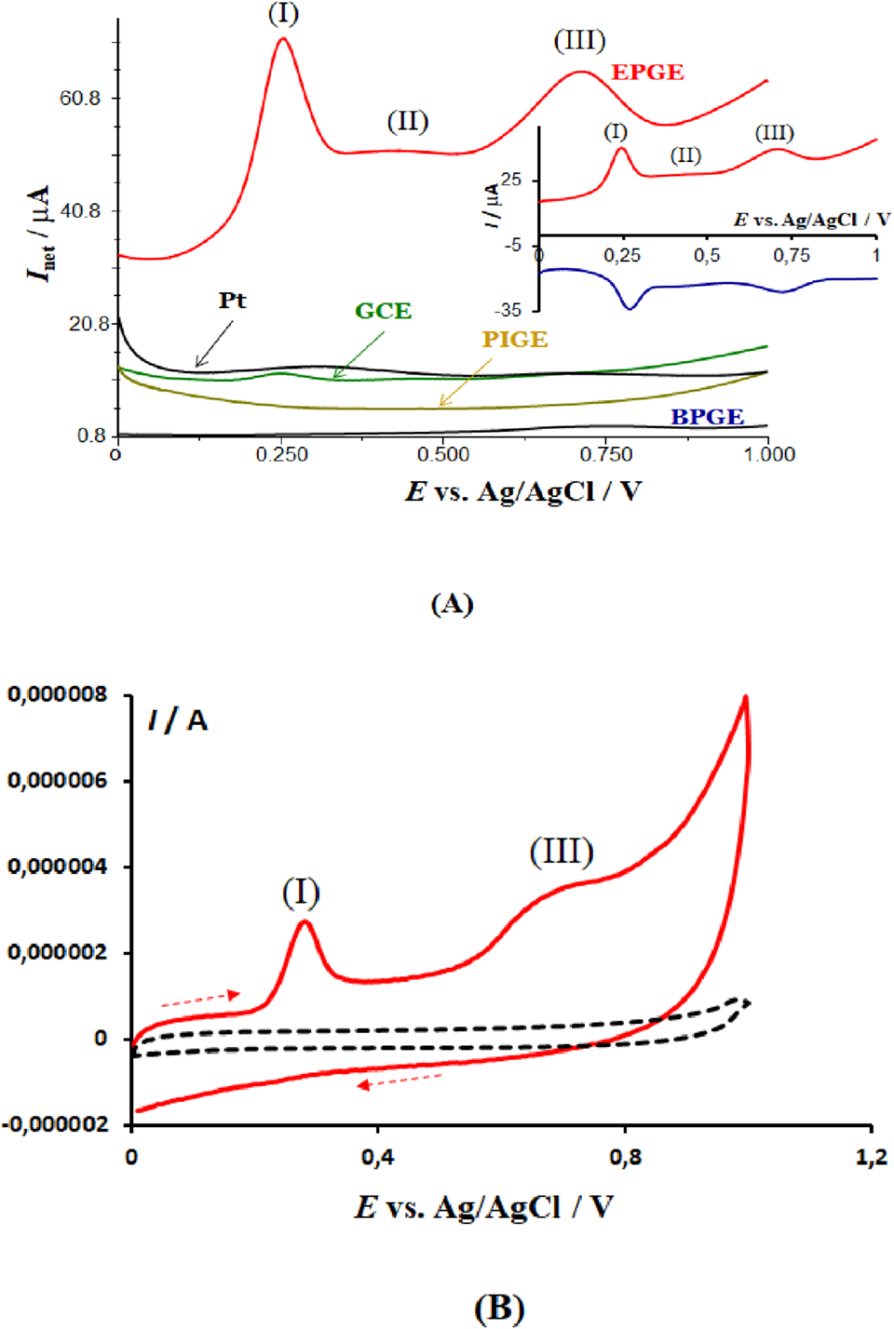
The panel (**A**) depicts the net square-wave voltammograms recorded at various working electrodes in a 20 mL phosphate buffer solution with a pH of 7.34 and containing 100 μL of human blood serum. The parameters of the potential modulation are as follows: SW frequency of 50 Hz, amplitude of 50 mV, and step potential of 1 mV. The inset displays the anodic (in red) and cathodic (in blue) voltammetric components, which correspond to the net voltammogram recorded at EPGE. The panel (**B**) also shows typical cyclic voltammograms of the phosphate buffer (represented by a dashed line) and the buffer containing 600 μL of human blood serum, scanned at a rate of 40 mV/s.

The voltammetric outcome of the blood serum is significantly influenced by several factors, including the type of working electrode, the pH of the buffer, the accumulation time, the potential applied prior to the voltammetric scan, and the type of voltammetric technique. The role of the electrode type is demonstrated in Fig. 1A, which displays the typical net SW voltammetric results obtained with Pt, GCE, PIGE, BPGE, and EPGE electrodes. Except for EPGE, all electrodes produced only a barely measurable voltammetric response from the serum sample of a healthy individual. However, EPGE showed a superior voltammetric profile with three typical net SW voltammetric peaks located near 0.250 V (I), 0.400 V (II), and 0.720 V (III) (refer to Fig. 1A). The intensity of process (II) is frequently very weak, while process (I) dominates in all serum samples.

In comparison, the morphology of the voltammetric profile under cyclic voltammetry (CV) conditions is less favourable when compared to square wave voltammetry (SWV) (Fig. 1B). A typical cyclic voltammogram reveals two irreversible processes, namely processes (I) and (III). Additionally, repetitive cycling of the potential significantly reduces the intensity of the response. Specifically, in the second potential scan, the anodic voltammetric peaks are barely visible, indicating electrode surface fouling^33^. Under SWV conditions, due to the speed of the technique, the blocking of the electrode surface is less significant. Nevertheless, it is important to thoroughly clean the electrode surface before each voltammetric scan, as described in the experimental section. Another notable difference from CV is the electrochemical reversibility of the electrode processes; under SWV conditions, all three electrode processes appear to be chemically reversible and electrochemically quasi-reversible, with well-developed anodic and cathodic SW voltammetric components, as shown in the inset of Fig. 1A.

**Figure 2:**
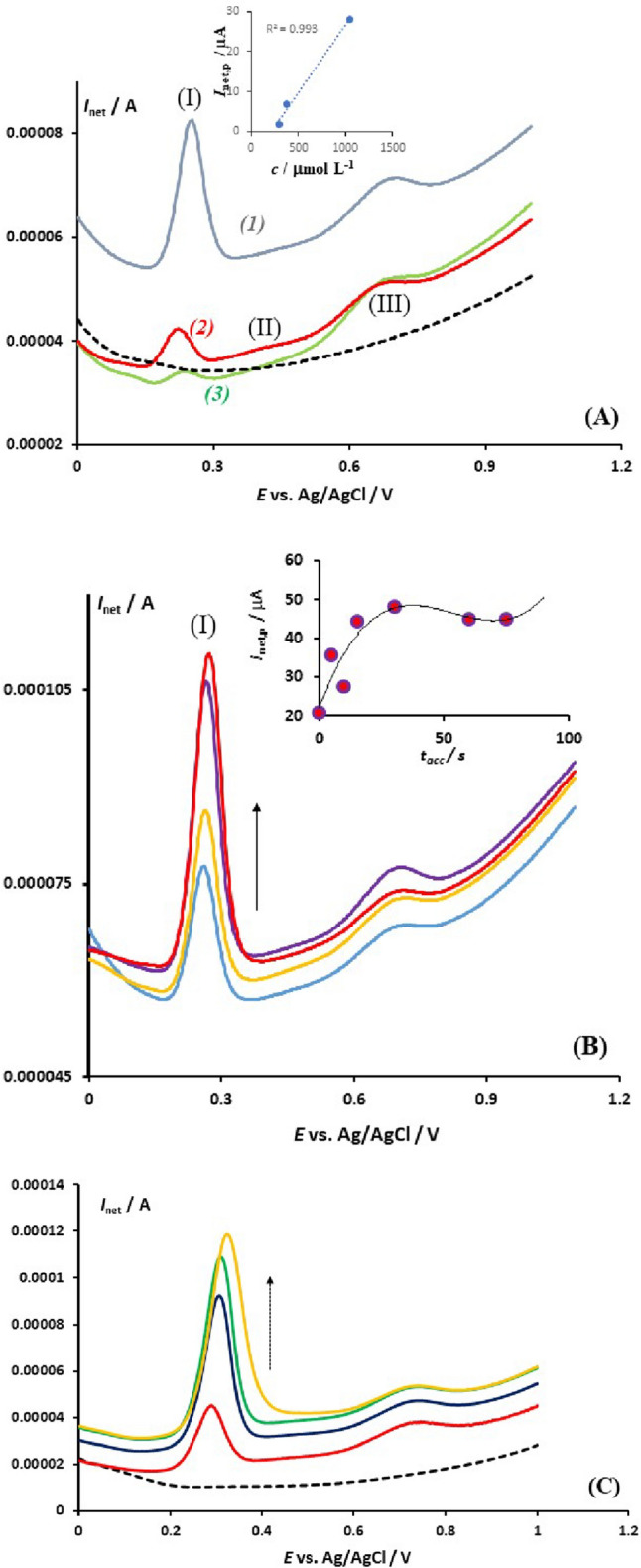
The figure illustrates the results of the net SW voltammograms recorded at the EPGE for different serum samples in a 20 mL phosphate buffer solution with a pH of 7.34 and containing 600 μL of the serum. The SW frequency was 25 Hz, the amplitude was 50 mV, and the step potential was 2 mV. The voltammogram of the blank buffer solution is shown as a dashed line. The inset in panel (**A**) displays the correlation between the net peak current of the process (I) and the concentration of uric acid (UA) in the serum sample. The concentrations of UA in the voltammetric cell were estimated to be 31.4 μmol/L (sample 1), 11.4 μmol/L (sample 2), and 9.0 μmol/L (sample 3) using independent methods in the biochemical clinical laboratory. In panel (**B**), the effect of the accumulation time on the intensity of the peak (I) of sample 1 is shown. The accumulation time increases in the direction of the arrow from 0, 10, 15 to 30 s, conducted at the initial potential of 0.00 V in a quiet solution. The inset in this panel displays the isotherm-like dependence of the net peak current (I) on the accumulation time. In panel (**C**), the effect of adding a standard solution of UA is shown. The dashed line corresponds to the supporting electrolyte (blank) and the red curve represents the serum sample where the UA in the voltametric cell was estimated to be 14.2 μmol/L. The other voltammograms were recorded after adding a standard solution of UA to the serum sample, where the total concentration of UA in the voltammetric cell increases in the direction of the arrow from 64.2, 114.2, and 164.2 μmol/L.

By comparing the typical net SW voltammograms of different serum samples, it becomes evident that the voltammetric response is highly sensitive to the composition of the serum (Fig. 2A). The curve labelled as (1) in Fig. 2A corresponds to the serum with the highest UA content (*c*[UA] = 1046 μmol/L) among the other samples. The intensity of peak (I) is directly correlated with the UA concentration, as demonstrated in the inset of Fig. 2A. Additionally, the intensity of peak (I) depends significantly on the accumulation time, which suggests a surface electrode mechanism (Fig. 2B). The net peak-current of process (I) follows an isotherm-like relationship with the accumulation time, as shown in the inset of Fig. 2B. UA effectively adsorbs on the electrode surface, competing with other surface-active components in the complex serum sample.

Under the conditions of Fig. 2B, with UA concentration of 31.4 μmol/L in the voltammetric cell, the electrode becomes saturated with adsorbed UA after about 30 s. Importantly, during the accumulation process, the response remains well-defined, with a constant peak potential and half-peak width (refer to Fig. 2B). As demonstrated in Fig. 2C, adding a standard solution of UA to the supporting electrolyte that contains a serum sample clearly increases the response of peak (I), confirming the correct assignment of the voltammetric peak to the electrode reaction of UA.

**Figure 3:**
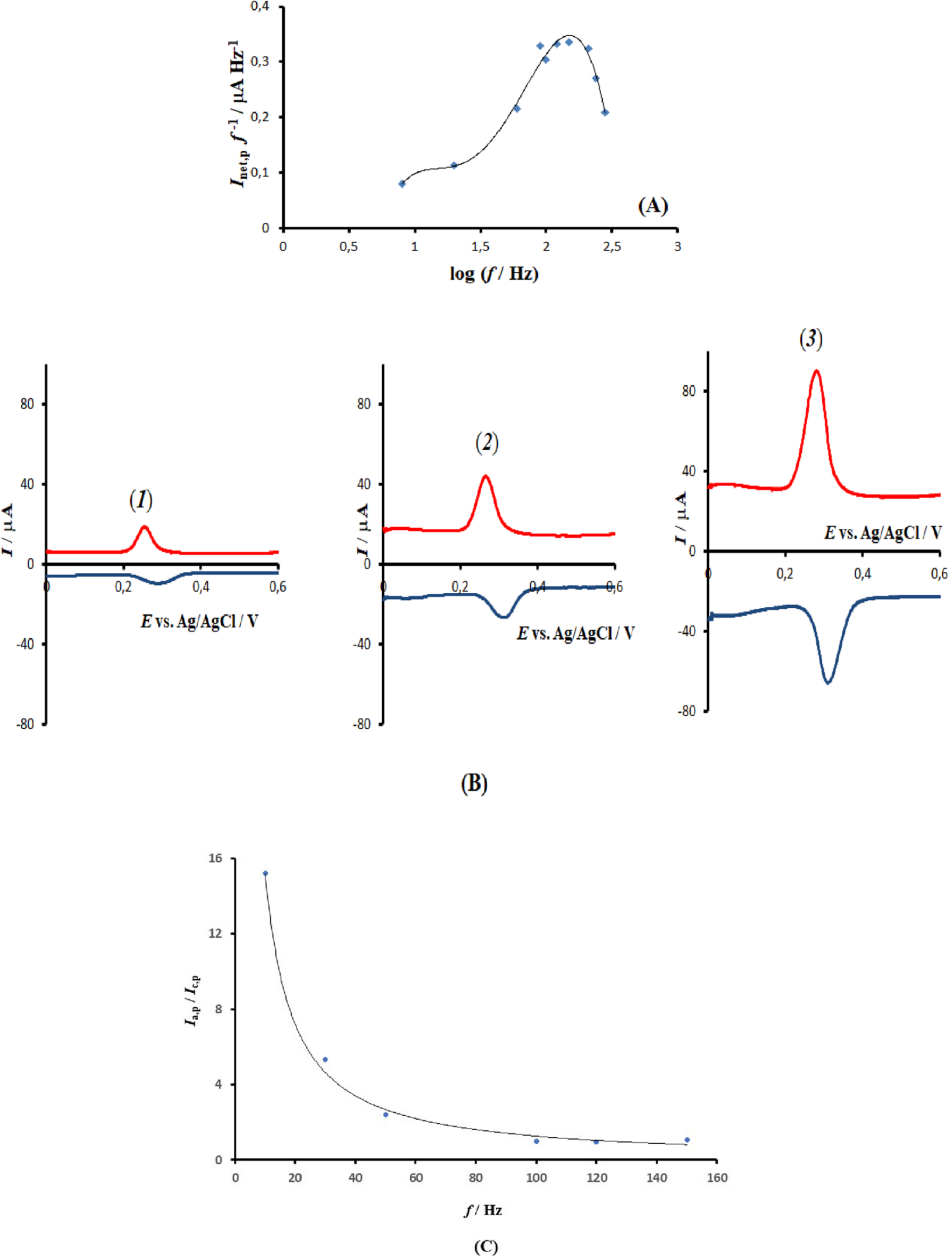
Analysis of the effect of SW frequency on voltammetric features of the process (I) at EPGE. (**A**) The relationship between frequency-normalized net peak current and the logarithm of the frequency. (**B**) Evolution of the anodic (red) and cathodic (blue) SW voltammetric components after 90 s of accumulation at a starting potential of 0.00 V for frequencies of 20 Hz (1), 60 Hz (2), and 100 Hz (3). (**C**) Dependence of the peak-current ratio of the anodic and cathodic SW voltammetric components on frequency. All other conditions are identical to those in Fig. 1.

Further examination of electrode reaction (I) was conducted by exploring the impact of the square-wave frequency and by closely observing the progression of all three voltammetric components in the square-wave voltammograms. It is important to note that a clear response can be obtained from any serum sample using a moderate square-wave frequency (*f*), typically below 250 Hz. At higher frequencies, however, the response is poorly defined, with hardly measurable voltammetric parameters, most likely due to complex adsorption phenomena that greatly affect charging and background currents, slow electron transfer at the electrode–electrolyte interface, and uncompensated resistance, all of which significantly impact surface electrode processes at higher square-wave frequencies^34^. Nonetheless, for *f* ≤ 250 Hz, the evolution of the voltammetric profiles aligns well with theoretical predictions for a quasi-reversible surface electrode reaction, despite the complexity of the medium. Specifically, the net peak-current of process (I) increases non-linearly with *f*, while the frequency-normalized net peak-current (*I*_net,p_/*f*) follows a parabolic relationship with log(*f*), resulting in the well-known characteristic of a "quasi-reversible maximum" (Fig. 3A)^4^. The critical frequency value related to the maximum position reflects the electrochemical standard rate constant, which is close to 150 s^-1^.

A close examination of the relationship between the anodic and cathodic components of the SW voltammetric response for various frequencies provides further insight into the mechanism of the electrochemical oxidation of UA. The SW voltammograms shown in Fig. 3B indicate that the intensity of the cathodic components increases relative to the anodic components with increasing frequency. The plot of the peak-current ratio of the anodic and cathodic components (*I*_a,p_/*I*_c,p_) in Fig. 3C reveals that the ratio decreases almost exponentially as the frequency increases. At a frequency of 150 Hz, the ratio is close to 1 and the intensities of the anodic and cathodic components are nearly equal. This voltammetric behaviour suggests that the oxidation of UA follows an EC reaction scheme, where the initial electron transfer reaction (E) is followed by a chemical reaction (C) that produces a redox-inactive product^35^.

**Figure 4:**
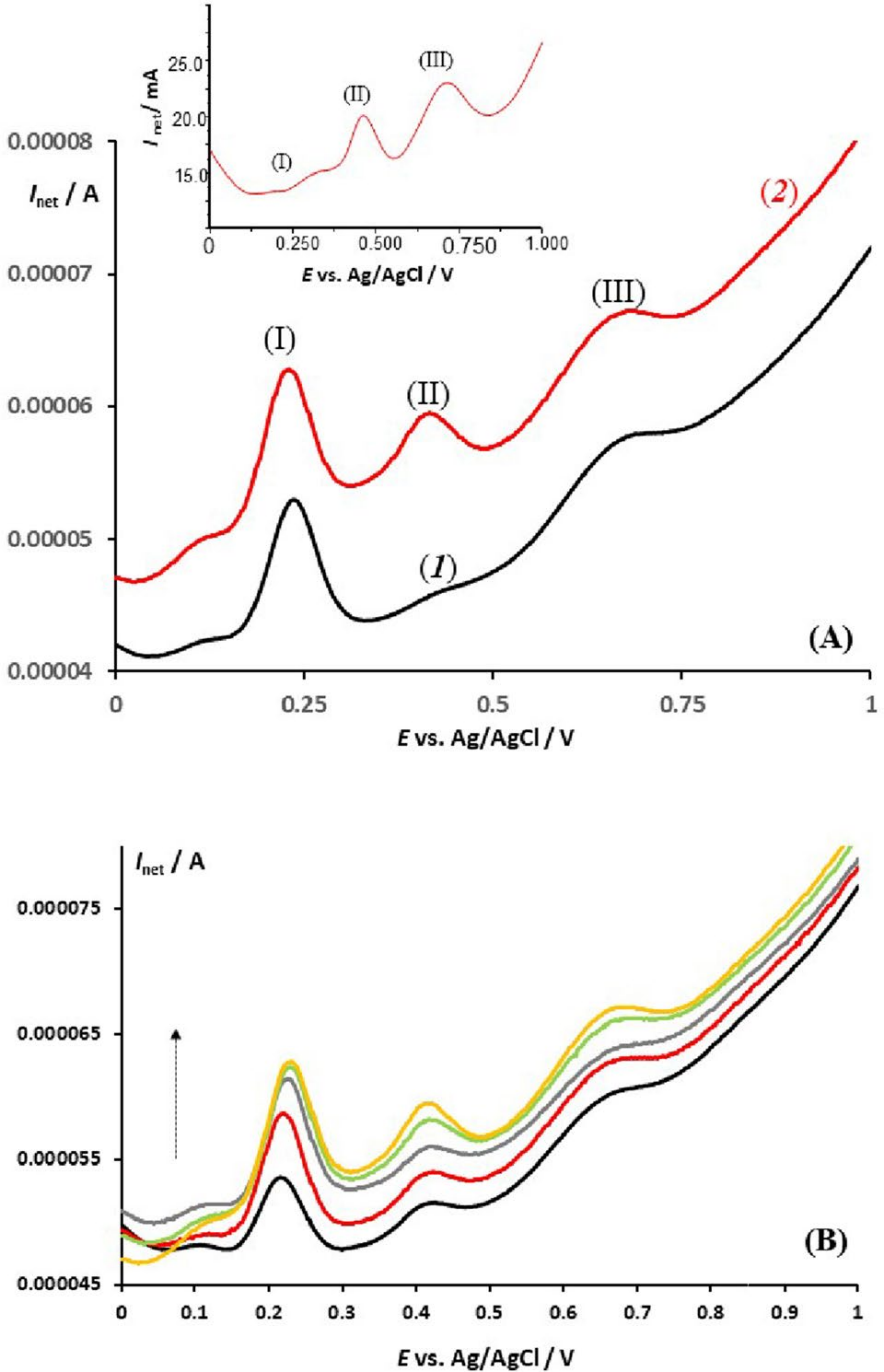
(**A**) The net SW voltammograms of a serum sample containing 3.9 μmol/L total BR (curve 1) and an additional spike of 1.25 μmol/L of standard BR solution (curve 2) are recorded at an EPGE with an accumulation time of 90 s at an initial potential of 0.00 V. The other conditions are identical to those in Fig. 2. The inset shows a typical voltammogram of a serum sample with elevated BR content (*c*(BR) > 600 μmol/L), recorded without accumulation at a SW frequency of 50 Hz. (**B**) The effect of accumulation time on the serum sample containing 11.38 μmol/L UA and 50.39 μmol/L BR. The accumulation time increases from 5 to 30 s in the direction of the arrow and is conducted at a potential of 0.00 V in a quiet solution. The other conditions are identical to those in Fig. 2.

Our further attention was focused on process (II), which typically exhibits low intensity in voltammograms of healthy individuals’ serum samples. Figure 4A presents characteristic voltammograms of serum samples with elevated BR content. Panel A refers to a sample containing 3.9 μmol/L in the voltammetric cell (curve 1). The peak II becomes measurable after a 90 s accumulation at the initial potential without any steering to the solution. Upon the addition of 1.2 μmol/L standard BR solution, the peak II clearly increases, confirming that process II is associated with the electrode reaction of BR (curve 2). The intensity of process II is also clearly visible in another serum sample containing a very high concentration of BR, as shown in the inset of Fig. 4A. With such a high concentration of BR, the response of UA is greatly reduced (peak I in the inset of Fig. 4A), indicating that competitive adsorption between UA and BR (and likely other species in the serum sample) plays a significant role in shaping the overall voltammetric response. To study this competitive adsorption further, we analysed a serum sample containing elevated concentrations of both UA and BR (Fig. 4B). When both components are present at comparable concentrations (i.e., *c*(UA) = 11.38 μmol/L and *c*(BR) = 50.39 μmol/L), both characteristic peaks I and II increase concurrently with the accumulation time. However, the adsorption of UA is more effective and produces a larger net peak-current at lower concentrations compared to BR.

**Figure 5:**
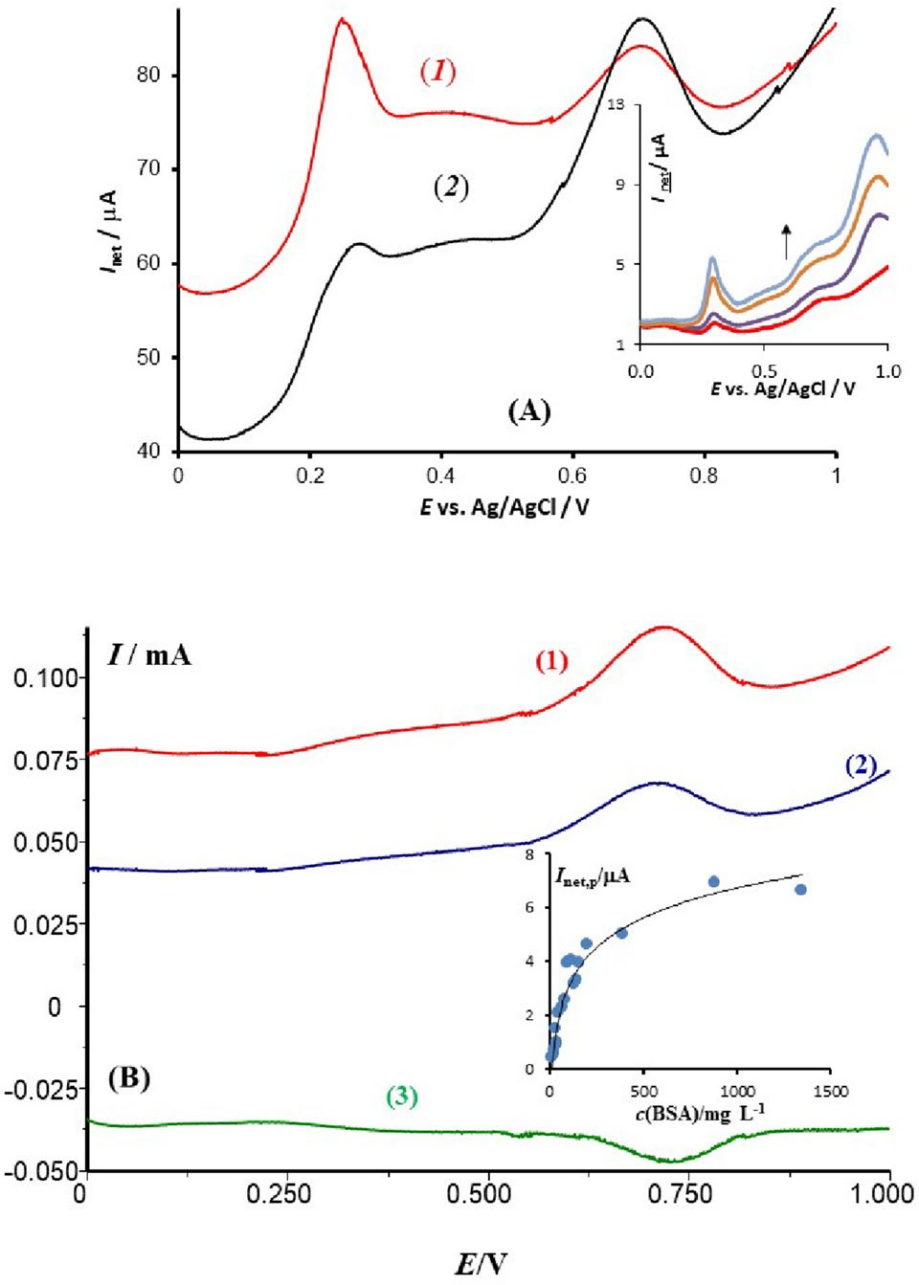
(**A**) Net SW voltammograms recorded at the EPGE working electrode in a 20 mL phosphate buffer at pH = 7.34, containing 200 μL of a serum sample from a healthy individual (1) and the same sample spiked with a standard solution of bovine serum albumin (2). The mass percentage of bovine serum albumin added to the cell is 20%. The parameters of the potential modulation are SW frequency of 20 Hz, amplitude of 50 mV, and step potential of 1 mV. The inset shows the response of a healthy individual serum sample (red curve) spiked with the standard of human serum albumin at concentration of 3.6, 7.6 and 9.0 μmol/L (in the arrow direction) recorded at the frequency of 50 Hz. (**B**) A typical SW voltammogram of bovine serum albumin at a concentration of 1350 mg/L, showing the net (1), anodic (2), and cathodic (3) voltammetric components recorded at a frequency of 100 Hz, SW amplitude of 50 mV, and step potential of 1 mV. The inset displays the dependence of the net peak current on the concentration of bovine serum albumin, recorded at a frequency of 50 Hz.

Our attention was drawn to the voltammetric peak III, which showed the most complex behaviour among peaks I and II. This peak appeared in all serum samples and was highly sensitive to both the accumulation time and the concentration of serum in the electrolyte solution. The results in Fig. 5A indicate that the peak III increased upon the addition of bovine serum albumin (BSA) standard solution, as well as the human serum albumin (HSA) (inset of Fig. 5A). Typically, increasing the accumulation time and the concentration of BSA in the sample caused the overall SW net voltammogram to shift towards lower absolute currents, implying that serum albumins adsorption was a critical factor in the overall background current^36,37^. An analogous voltammetric behaviour was also observed with HSA; however, the HSA standard solution contains stabilizing compounds (e.g., sodium azide etc.) which gives rise to a strong interfering voltammetric peak at about 0.900 V (the inset of Fig. 5A), thus precluding further experiments with HSA. By conducting experiments with BSA only, one could imply that peak III corresponded to the electrode reaction of albumins. The broad net SW peak at approximately 720 mV is a result of a slow, quasi-reversible electrode reaction, which is consistent with the data in Fig. 1A (as seen in the inset of Fig. 1A). The intensity of the net peak was dependent on both the serum albumins concentration and the accumulation time. The inset of Fig. 5B shows the dependence of the net peak-current on the BSA concentration, highlighting the adsorption characteristics of BSA and the saturation of the electrode at concentrations above 500 mg/L.

## Discussion

The experimental data presented above demonstrate the effectiveness of SWV in providing insight into the electrochemistry of a variety of species in a complex medium when applied at a macroscopic electrode with superior electrocatalytic properties such as EPGE, which is a commercially available and conventional electrode. Despite numerous analytical studies aimed at detecting specific small biomolecules or drugs in a serum sample^38–40^, this study is the first to use SWV in a spectroscopy-like manner to assign the voltammetric profile of a serum sample to the dominant underlying electrode processes. It is worth mentioning that attempts to study blood serum voltammetrically date back to the early works of Koryta et al. ^5,6^, where CV was applied at a Pt electrode. More recent studies have used CV to detect antioxidants or to generally characterize the redox status of a serum^41,42^. However, when comparing the typical SW voltammetric profile of a serum sample (red curve in Fig. 1A) with the corresponding cyclic voltammogram (red solid curve in Fig. 1B), the superiority of SWV in terms of resolution of the voltammetric peaks, response intensity, and electrochemical reversibility of the underlying electrode processes is evident. In addition to the importance of the voltammetric technique, the results depicted in Fig. 1A emphasize the crucial role that the type of working electrode plays in achieving a clear and reproducible voltammetric profile in highly complex media such as blood serum. The serum contains more than 300 different proteins, in addition to glucose, lipids, electrolytes, and a variety of exogenous substances. Previous studies^43–45^, have shown that EPGE is less prone to fouling and passivation, and its high density of edge plane sites allows for effective adsorption of small molecules such as UA and BR (as demonstrated in Figs. 2 and 4).

The accumulation time study presented in Figs. 2B and 4B confirms that the concomitant adsorption of proteins, including albumin^46^ does not hinder the accumulation and electrochemical activity of both UA and BR. Despite the overall voltammetric profile increasing with accumulation time due to protein adsorption, the abundant edge plane sites of the EPGE remain largely free for UA and BR adsorption. Importantly, in a sample containing comparable concentrations of UA and BR, both molecules adsorb on the electrode surface without significant interference, as shown in Fig. 4B. It is worth noting that the response of BR is only clearly visible in samples with a higher BR content relative to the concentration in a healthy person’s serum, meaning that the concentration in the voltammetric cell should be higher than 0.3 µmol/L.

The typical SW voltammograms of a serum are impacted by adsorption as depicted in Figs. 2B, 4B, and 5B. This is further demonstrated through the data in Fig. 3, which pertains to the electrode mechanism of UA. The SW voltammograms of uric acid oxidation recorded in such a complex matrix are consistent with theoretical predictions for an idealized surface electrode mechanism^47^.

The morphological analysis of the voltammetric profile presented in Fig. 3B, considering the peak-like shape of the forward (anodic, oxidative) and backward (cathodic, reductive) SW voltammetric components and their positioning on the potential axis, is characteristic of a surface electrode reaction where both components of the redox couple are fixed on the electrode surface^4^. The relative positioning of the anodic voltammetric component at more negative potentials than the corresponding cathodic component (Fig. 3B), recorded at a moderate SW amplitude of 50 mV, aligns with theoretical predictions for fast electrode processes of a firmly attached redox couple where mass transfer is negligible during the voltammetric scan^4^. By analysing the net peak-current for different SW frequencies (i.e., varying durations of potential pulses), the "quasi-reversible maximum" is obtained (Fig. 3A), which has been theoretically predicted for various surface electrode processes^4^. The frequency-normalized net peak current (*I*_net,p_/*f* in Fig. 3A) reaches its maximum value for a given frequency, implying a synchronization of the electron transfer rate with the duration of the SW potential pulses^4^. Based on the position of the quasi-reversible maximum, the standard rate constant can be estimated as *k*_s_ = 150 s^-1^, assuming an electron transfer coefficient of *α* = 0.5 and a stoichiometric number of electrons of *n* = 2^4^.

Further understanding of the electrode mechanism of UA can be achieved by examining the relative intensities of the anodic and cathodic SW voltammetric components for different SW frequencies (i.e., varying durations of pulses and thus scan rates). When the frequency is low and the scan rate of the experiment is slow (panel 1 in Fig. 3B), the cathodic component is significantly less intense compared to the anodic component. Conversely, when the experiment is conducted at a relatively fast frequency of 100 Hz and short pulse duration (*t*_p_ = 1/(2*f*) = 5 ms^4^), the intensity of the cathodic component is almost equal to that of the anodic component (panel 3 in Fig. 3B). A detailed analysis of the anodic-to-cathodic peak current ratio (*I*_a,p_/*I*_c,p_) as a function of frequency is presented in Fig. 3C, revealing that the ratio decreases with increasing frequency as a result of the relative increase of the cathodic component caused by the shortening of pulse duration. This voltammetric behaviour is typical of a surface EC-type electrode mechanism where the electrode reaction (E) is followed by an irreversible chemical reaction (C), yielding an electro-inactive final product ^35^. This aligns with the mechanism of UA, where two-electron-two-proton oxidation (E step) is followed by a fast hydration reaction (C step), yielding allantoin as the final electro-inactive product^12,48^.

The recent analysis highlights notable disparities in the electrochemical reversibility observed under cyclic voltammetry conditions (Fig. 1B) and square-wave voltammetry (as shown in the inset of Figs. 1A and 3B). The high velocity of SWV makes the oxidation process of UA appear nearly reversible, as the product of electrochemical oxidation can be electrochemically reduced back to the native form of UA before it is entirely consumed by the spontaneous subsequent hydration reaction. On the other hand, the slower CV experiment leads to full consumption of the electrochemical product, resulting in irreversible voltammetric behaviour.

A close examination of the voltammetric responses of BR and ALB suggests that both processes are also nearly reversible (as demonstrated in the inset of Figs. 1A and 5B). In agreement with previous findings, the electrode mechanism of BR is of an EC type^35^; therefore, similarly to UA, the fast pace of the SWV reduces the impact of the follow-up chemical reaction, making the process nearly reversible. The electrode reaction pathway of ALB remains unknown, but it is presumably related to the oxidation of side amino acids^49^. Our comparative voltammetric data obtained through SWV (Fig. 5B) and CV (Fig. 1B) indicate an EC type electrode mechanism for ALB as well.

The nearly reversible nature of the electrode processes of UA, BR, and ALB is essential for the shape of the overall net SW voltammetric response, specifically in terms of strong signal intensity (and thus, improved analytical sensitivity) and narrow half-peak width (and thus, higher resolution of the voltammetric peaks). Furthermore, the fast voltammetric experiment and short electrolysis time minimize the fouling of the electrode by the products of the specific electrode reaction^37^.

## Conclusions

It is demonstrated for the first time that square-wave voltammetry can be employed as a sort of spectroscopic technique for fast, reliable, and direct electrochemical characterization of human blood serum samples without any pre-treatment of the sample or the surface of the edge plane pyrolytic graphite electrode. The quality of the voltammetric response is superior compared to literature data, with respect to the intensity, resolution, and electrochemical reversibility of the voltammetric peaks. The data suggest that the edge plane sites of the electrode are critically important for the effective adsorption, particularly of uric acid and bilirubin, as the response is virtually absent when a range of other electrodes are used. Accordingly, it is plausible to expect that the edge side of carbon nanotubes can provide a similar voltammetric response. The presented collection of experimental data is expected to provide a basis for the further development of electrochemical sensors for direct detection and quantification of these analytes in a buffered blood serum sample.

## Data Availability

The datasets produced and/or analysed during the present study are not publicly accessible due to the confidentiality of participants' personal information. However, they can be obtained from the corresponding author valentin.mircheski@chemia.uni.lodz.pl upon reasonable request.
